# Factors associated with recruitment, surveillance participation, and retention in an observational study of pregnant women and influenza

**DOI:** 10.1186/s12884-019-2280-0

**Published:** 2019-05-08

**Authors:** Mark G. Thompson, De-Kun Li, Allison L. Naleway, Jeannette R. Ferber, Michelle L. Henninger, Pat Shifflett, Leslie Z. Sokolow, Roxana Odouli, Tia L. Kauffman, Rebecca V. Fink, Joanna Bulkley, Janet D. Cragan, Sam Bozeman

**Affiliations:** 1 Influenza Division, Centers for Disease Control and Prevention (CDC), Atlanta, GA USA; 2 Division of Research, Kaiser Foundation Research Institute, Oakland, CA USA; 3 Department of Health Research and Policy, School of Medicine, Stanford University, Palo Alto, CA USA; 4 Center for Health Research, Kaiser Permanente Northwest, Portland, OR USA; 5 Abt Associates, Inc., Cambridge, MA USA; 6 Battelle Memorial Institute, Atlanta, GA USA; 7 National Center on Birth Defects and Developmental Disabilities, CDC, Atlanta, GA USA

**Keywords:** Cohort, Bias, Pregnant women, Influenza, Influenza vaccine effectiveness, Acute respiratory illness, Surveillance, Retention

## Abstract

**Background:**

This report describes the results of recruitment efforts and the subsequent participation of pregnant women in study activities in a 2010–2012 observational study focused on influenza illness and vaccination in California and Oregon, USA.

**Methods:**

Socio-demographic and health characteristics extracted from electronic medical records were compared among pregnant women who enrolled in the study, refused to participate, or were never reached for study invitation. These characteristics plus additional self-reported information were compared between women who enrolled in two study tracks: a prospective cohort vs. women enrolled following an acute respiratory illness (ARI) medical encounter. The characteristics of women who participated in weekly ARI surveillance (cohort enrollees, year one) and a 6-month follow-up interview (all enrollees) were also examined.

**Results:**

In year one, we reached 51% (6938/13,655) of the potential participants we tried to contact by telephone, and 20% (1374/6938) of the women we invited agreed to join the prospective cohort. Women with chronic medical conditions, pregnancy complications, and medical encounters for ARI (prior to pregnancy or during the study period) were more likely to be reached for recruitment and more likely to enroll in the cohort. Twenty percent of cohort enrollees never started weekly surveillance reports; among those who did, reports were completed for 55% of the surveillance weeks. Receipt of the influenza vaccine was higher among women who joined the cohort (76%) than those who refused (56%) or were never reached (54%). In contrast, vaccine uptake among medical enrollees in year one (54%; 53/98) and two (52%; 79/151) was similar to other pregnant women in those years. Study site, white race, non-Hispanic ethnicity, and not having a child aged < 13 years at home were most consistently associated with joining as a cohort or medical enrollee and completing study activities after joining.

**Conclusions:**

We observed systematic differences in socio-demographic and health characteristics across different levels of participant engagement and between cohort and medical enrollees. More methodological research and innovation in conducting prospective observational studies in this population are needed, especially when extended participant engagement and ongoing surveillance are required.

**Electronic supplementary material:**

The online version of this article (10.1186/s12884-019-2280-0) contains supplementary material, which is available to authorized users.

## Background

Prospective, community-based studies of the epidemiology of infectious diseases and the effectiveness of treatment and prevention strategies including vaccination are rare, in large part due to their high costs and many logistical challenges [1, 2]. Yet, observational studies are essential for understanding the clinical presentation of infectious diseases, transmission dynamics, and the effectiveness of interventions, in particular for populations at high-risk of morbidity and mortality such as pregnant women. However, there is a dearth of information on how best to conduct cohort and active surveillance studies among pregnant women [3–5].

This lack of information was especially evident to us in 2009 as we planned a two-year observational study of influenza vaccine effectiveness (IVE) in preventing laboratory-confirmed influenza illness during pregnancy [5]. Although we successfully completed the study and have previously reported IVE [6] and other findings on the epidemiology and prevention of influenza during pregnancy [7–10], many of the planning assumptions we made about cohort recruitment and surveillance participation [5] were proven wrong. Therefore, after completing the main study objectives, we returned to take a closer look at how pregnant women responded to our study requests and consider possible sources of selection and information bias.

This report describes the results of our recruitment efforts and the subsequent participation of pregnant women in study activities, including a 6-month post-delivery follow-up interview. The main project consisted of three sub-studies. In both years, we recruited pregnant women following medical encounters for acute respiratory illness (ARI) during influenza season; the women in these sub-studies are referred to as *medical enrollees (year one)* and *medical enrollees (year two)*. Because of uncertainty regarding where and how often pregnant women sought medical care for ARI, we also recruited pregnant women prospectively into a cohort prior to the initiation of influenza season in year one; this also gave us an opportunity to examine a broader range of illness severity. Participants in this part of the study are referred to as *cohort enrollees (year one)*.

Because our study took place within two integrated healthcare systems with longitudinal electronic medical record (EMR) data, we had the unique opportunity to identify the demographic and health characteristics of women who were eligible for our study (as either a medical or cohort enrollee) but could not be reached for recruitment, as well as women who were contacted but refused participation. Specifically, we examined possible indications of selection bias, such as systematic differences in age, study site, race, ethnicity, underlying health status, and medical visits. Given the focus of the original study on IVE, we were particularly interested in whether receipt of influenza vaccination differed between participants and non-participants and between the cohort vs. medical enrollee sub-studies. We also examined the associations between these factors and participation in weekly surveillance (for cohort enrollees in year one) and completion of a follow-up interview (among all enrollees in both years).

## Methods

### Study overview

We have previously published detailed methods of the study [5, 6]. In brief and relevant to the current analyses, participants were members of one of two separate regional integrated healthcare systems who attended at least one prenatal visit.

### Cohort enrollee recruitment

The year one cohort recruitment methods differed slightly by site. Both sites distributed flyers about the study to women following their prenatal visits via postal and electronic mail (at site one) or directly at prenatal and laboratory appointments (at site two). Information on the flyer gave women the opportunity to accept or decline participation immediately by mail or telephone. For all other women who did not immediately accept or decline participation, both sites then called potential participants on the telephone; this was done by local staff at site one and by an external call center at site two. Verbal orientation to the study and completion of verbal consent were completed during the telephone contact, and signed written consent forms were returned by postal mail or during the home visit when enrollment interviews were completed by study staff.

### Medical enrollee recruitment

During both years one and two, we recruited pregnant women during the influenza season following medical encounters for ARI (defined below); these medical enrollees were recruited by telephone at both sites. In year one, medical enrollees could only be recruited from women not already participating in the cohort. Completion of the enrollment interview and collection of respiratory specimens were done during a home visit by study staff. In year two only, we also recruited two pregnant women controls for each laboratory-confirmed influenza positive pregnant woman enrolled. The control participants were recruited by telephone within 2 weeks of each influenza positive case, were in the same trimester of pregnancy, and had no record of a medical encounter for ARI and no self-report of an illness with fever and cough since the start of the local influenza season. These women are referred to as *ARI-negative controls.* Medical enrollees and ARI-negative controls provided verbal consent when recruited by telephone and then written consent by postal mail or when they met with study staff.

### Measures

Ten variables were available from the EMR for all potential participants. Most indicators describe the women’s characteristics or medical history at the time of (or prior to) recruitment: (a) socio-demographic characteristics (study site, white race, Hispanic ethnicity, and the woman’s age and days of gestation at the start of the influenza season); (b) presence of a high-risk chronic medical condition (associated with increased risk of influenza complications; codes available from the authors) during the year prior to conception; (c) medical encounters for an ARI during the year prior to conception (using International Classification of Diseases, Ninth Revision, Clinical Modification [ICD-9 CM] codes 460–466 and 480–488). Other indicators describe women’s medical history during the entire pregnancy or study period and thus enable us to examine how women who differed in participation may have differed in their health trajectories: (d) ARI medical encounters during the 6-month study period; (e) influenza vaccination (at any point during the Fall or Winter of the study year); (f) any pregnancy complications during the index pregnancy (using a subset of the ICD-9-CM codes 640–649 related to adverse pregnancy outcomes [9]).

Additional self-reported variables were available for cohort enrollees and medical enrollees (including ARI-negative controls) who completed interviews at enrollment: (a) expanded race categories; (b) marital status; (c) education; (d) subjective social status (SSS) rated on a 9-point ladder from low (1) to high (9) and examined in tertiles (see [11–13]); (e) number of children aged < 13 years living at home; (f) overall self-rated health (SRH) status [13, 14]; (g) smoking status currently or prior to pregnancy; (h) presence of 17 stressful events in the year prior to the interview (as previously described [15]).

### Comparisons of enrollees, refusers, and never reached potential participants

The comparisons we were able to make differed for the two years of our study. For year one (see columns of Table 1), we compared the characteristics of cohort enrollees with medical enrollees; then we combined these two groups of consented participants and compared their characteristics with women who *refused* to participate in the study when invited. Finally, we compared the characteristics of women we called to invite to join the study but who were *never reached* with women who we reached (including those who consented or refused). Because ethnicity data was incomplete for non-participants, the available data is presented for refusers and never reached women, but statistical comparisons are not made.

**Table 1:** Characteristics of medical enrollees, cohort enrollees, participation refusers, and those never reached for cohort invitation in study year one (2010–11)

	A	B	A vs. B	C	A + B vs. C	D	A + B + C vs. D
	Cohort Enrollees	Medical Enrollees ^a^	Medical vs. Cohort Enrollees	Reached but Refused to Participate	Medical and Cohort Enrollees vs. Refused	Never Reached for Participation Invite	Never Reached vs. Reached (Enrolled + Refused)
Total N	1374	98		5564		6717	
Source: Medical Record, Categorical Variables, N (Col. %)							
Study Site			*p* < .0005		*p* < .0005		*p* < .0005
Site 1	565 (41)	16 (16)		1248 (22)		786 (12)	
Site 2	809 (59)	82 (84)		4316 (78)		5931 (88)	
Race (White)			*p* < .0005		*p* < .0005		*p* < .0005
White	934 (68)	46 (47)		3304 (59)		3822 (57)	
Non-White	440 (32)	52 (53)		2037 (37)		2689 (40)	
Unknown Race				223 (4)		206 (3)	
Ethnicity			*p* < .0005		NA ^b^		NA ^b^
Hispanic/Latina	173 (13)	27 (28)		1114 (20)		1802 (27)	
Non-Hispanic	1201 (87)	71 (72)		2213 (40)		2375 (35)	
Unknown Ethnicity				2237 (40)		2540 (38)	
High Risk Medical Conditions							
Chronic Condition (prior to pregnancy)	277 (20)	21 (21)	*p* = .76	581 (10)	*p* < .0005	731 (11)	*p* = .007
Pregnancy Complication	1034 (75)	89 (91)	*p* < .0005	3567 (64)	*p* < .0005	4292 (64)	*p* = .001
Medical visits for ARI (1 or more)							
Year Prior to Study (12 months)	320 (23)	38 (39)	*p* = .001	925 (17)	*p* < .0005	1075 (16)	*p* = .33
During Study Period (6 months)	307 (22)	98 (100)	*p* < .0005	953 (17)	*p* < .0005	850 (13)	*p* < .0005
Study Season’s Influenza Vaccination	1038 (76)	53 (54)	*p* < .0005	3029 (54)	*p* < .0005	3533 (53)	*p* < .0005
Medical Record, Continuous, Mean (95% CI)							
Woman’s Age (at start of season), Years ^c^							
Both Sites	31.7 (31.4–31.9)	29.9 (28.7–31.1)					
Site 1	30.6 (30.1–31.0)	28.1 (26.8–29.4)		29.1 (28.8–29.4)		28.9 (28.5–29.3)	
Site 2	32.4 (32.1–32.7)	30.3 (29.7–31.0)		31.4 (31.2–31.5)		31.4 (31.3–31.5)	
Gestational Age (at start of season), Days ^c^							
Both Sites	179 (176–182)	184 (172–196)					
Site 1	155 (151–160)	159 (146–172)		132 (127–138)		134 (127–141)	
Site 2	199 (195–202)	178 (171–185)		168 (166–173)		166 (164–168)	
Source: Enrollment Interview, Categorical, N (Col. %)							
Race (Self-Reported)			*p* < .0005				
White	934 (68)	46 (47)					
Asian	205 (15)	19 (19)					
Black	64 (5)	5 (5)					
Mixed or Other	171 (12)	28 (29)					
Marital Status			*P* = .009				
Married or Partnered	1286 (94)	85 (87)					
Not Married and Not Partnered	88 (6)	13 (13)					
Education			*p* < .0005				
High school or less	119 (9)	19 (19)					
Some college or bachelor’s degree	744 (54)	64 (65)					
Advanced degree	511 (37)	15 (15)					
Subjective social status			*p* = .002				
1 to 3 (low)	25 (2)	5 (5)					
4 to 6 (medium)	697 (51)	63 (64)					
7 to 9 (high)	632 (46)	28 (29)					
*Missing or refused*	20 (1)	2 (2)					
Child(ren) aged < 13 years at home			*p* = .049				
No	644 (47)	36 (37)					
Yes child	730 (53)	62 (63)					
Self-rated Health Status			*p* < .0005				
Poor, fair, or good	301 (22)	46 (47)					
Very good	548 (40)	32 (33)					
Excellent	525 (38)	20 (20)					
Smoking			*p* = .23				
Never smoked	1016 (74)	67 (68)					
Previously or currently smoke	358 (26)	31 (32)					
Stressful events in past year (0–17)			*p* = .11				
0	276 (20)	11 (11)					
1	349 (25)	24 (24)					
2	310 (23)	23 (23)					
3 or more	439 (32)	40 (41)					

*Abbreviations*: *NA* Not applicable, *ARI* Acute respiratory illness; ICD-9-CM codes 460–466 and 480–488

^a^Medical enrollees are women who were recruited by telephone following a medical visit for ARI

^b^We did not calculate a statistical comparison for ethnicity with the study refusers and never reached women given the large number with unknown ethnicity in the medical records; the comparison of medical vs. cohort enrollees relied on self-reported ethnicity

^c^Women’s age and age of fetus were calculated using a common reference date, which was the start of the influenza season at each study site. Age was calculated combining sites for fully enrolled women, but had to be calculated by study site for women not enrolled

For study year two (see columns for Table 2), we compared the characteristics of medical enrollees and their ARI-negative controls; then, we combined these two groups of consented participants and compared their characteristics with women who were invited to either group but refused participation. Unfortunately, for year two, we do not have records of women who we attempted to contact by telephone but never reached. Therefore, for year two, we can only compare enrollees with all other pregnant women during the study period (excluding those who refused participation).

**Table 2:** Characteristics of medical and control enrollees, those who refused participation, and all other pregnant women at study sites during study year two (2011–12)

	A	B	A vs. B	C	A + B vs. C	D	A + B vs. D
	Medical Enrollees (Year 2) ^a^	ARI-Negative Control Enrollees (Year 2) ^a^	Medical vs. Control Enrollees	Reached for Recruitment but Refused to Participate	Medical and Control Enrollees vs. Refused	All Other Pregnant Women During Study (Year 2) ^b^	*Enrollees* vs. *All Other Pregnant Women*
Total N	151	103		514		19,264	
Source: Medical Record, Categorical Variables, N (Col. %)							
Study Site			*p* < .0005		*p* < .0005		*p* < .0005
Site 1	50 (33)	20 (19)		229 (45)		3466 (18)	
Site 2	101 (67)	83 (81)		285 (55)		15,798 (82)	
Race (White)			*p* = .01		*p* = .01		*p* < .0005
White	81 (54)	39 (38)		309 (60)		11,213 (58)	
Non-White	79 (52)	64 (62)		178 (35)		7409 (38)	
Unknown Race				27 (5)		642 (3)	
Ethnicity			*p* = .045		NA ^c^		NA ^c^
Hispanic/Latina	53 (35)	24 (23)		96 (19)		3684 (19)	
Non-Hispanic	98 (65)	79 (77)		147 (29)		7206 (37)	
Unknown Ethnicity				271 (53)		8374 (43)	
High Risk Medical Conditions							
Chronic Condition (prior to pregnancy)	37 (25)	12 (12)	*p* = .01	95 (18)	*p* = .27	2108 (11)	*p* = .03
Pregnancy Complication	128 (85)	76 (74)	*p* = .03	380 (74)	*p* = .47	11,816 (61)	*p* = .001
Medical visits for ARI (1 or more)							
Year Prior to Study (12 months)	39 (26)	24 (23)	*p* = .21	133 (26)	*p* = .67	2799 (15)	*p* < .0005
During Study Period (6 months)	NA	NA		NA		NA	
Study Season’s Influenza Vaccination	79 (52)	65 (63)	*p* = .09	130 (25)	*p* = .008	7432 (39)	*p* = .17
Medical Record, Continuous, Mean (95% CI)							
Woman’s Age (at start of season), Years ^d^							
Site 1	28.5 (26.8–29.5)	28.5 (26.8–29.5)		30.0 (29.2–30.7)		29.4 (29.1–20.5)	
Site 2	30.9 (230.1–31.7)	30.9 (230.1–31.7)		31.3 (30.7–32.0)		31.4 (31.3–31.5)	
Gestational Age (at start of season), Days ^d^							
Site 1	105 (79–130)	105 (79–130)		123 (108–137)		146 (142–150)	
Site 2	97 (85–110)	97 (85–110)		91 (81–101)		105 (104–107)	

*Abbreviations*: *NA* Not applicable, *ARI* Acute respiratory illness; ICD-9-CM codes 460–466 and 480–488

^a^Medical enrollees are women who were recruited by telephone following a medical visit for ARI; ARI-negative controls had no medical visit for ARI and had not self-reported illness with fever and cough since the start of the local influenza season

^b^Records for potential participants who could not be reached by telephone were not available for study year 2; therefore, “all other pregnant women during study” includes women we attempted to contact but never reached and the much larger population of pregnant women who were not selected as potential participants

^c^We did not calculate a statistical comparison for ethnicity for refusers and the full source population given the large number with unknown ethnicity in the medical records; the comparison of medical vs. ARI-negative control enrollees relied on self-reported ethnicity

^d^Women’s age and age of fetus were calculated using a common reference date, which was the start of the influenza season at each study site

### Active surveillance

As described in previous reports [6, 7], year one cohort participants were asked to report weekly on their health (including illness symptoms like cough and fever) throughout the influenza season using 1 of 3 methods: (a) logging on to a central study website, (b) calling by telephone into a central interactive voice response system, or (c) calling staff at the study site directly. If a participant missed a surveillance report, study staff attempted to contact her by telephone and postal/electronic mail within a week. We calculated the percentage of weekly reports when a surveillance contact was completed, excluding weeks when the participant was sick with an ARI and thus was not expected to participate in surveillance. Because year two of the study focused on medical enrollees, there was no prospective cohort or active surveillance in year two.

### Statistical analysis

For comparisons involving women who were never reached or refused participation, local IRBs approved analyses of frequency tables rather than individual-level data; thus, we used Chi-square tests to compare counts of their socio-demographic and health characteristics (Tables 1 and 2). For comparisons of cohort and medical enrollees in year one and medical and ARI-negative control enrollees in year two, individual-level data were available; thus, in addition to bivariate comparisons using Chi-square tests, we completed multivariable tests using logistic regression in order to note which associations remained statistically significant (*p* < 0.05) when all variables were considered simultaneously.

## Results

### Cohort enrollees compared to non-participants in year one

Of 13,655 potentially eligible pregnant women that we attempted to recruit across sites for the prospective cohort during year one, we reached half by telephone (51%); a substantially higher proportion of women were reached at site one (70%, 1813/2599) where local staff made invitation calls, than at site two (46%, 5125/11,056) where an outside contractor made the calls. Similarly, successful completion of informed consent and enrollment in the cohort among invited women was also higher at site one (31%, 565/1813) than site two (16%, 809/5125); the combined enrollment rate was 20% (1374/6938) (Additional file 1: Figure S1).

As shown in Table 1, the following characteristics were significantly associated with recruitment and participation status (reached vs. never reached, and among those reached, enrolled vs. refused): Study site, white race, having a chronic medical condition prior to pregnancy, having at least one pregnancy complication during the pregnancy, having a medical encounter for ARI during the study period, and receiving the influenza vaccine for the study season. At the two sites, women who enrolled in the cohort were aged 0.7 and 1.0 year older than those who refused; cohort enrollees were also further along in their pregnancies by 123 and 51 days at the two sites.

### Cohort enrollees compared to medical enrollees in year one

The following characteristics were more common among *medical* enrollees (contacted following an ARI medical encounter) compared to the *cohort* enrollees in Year 1 (Table 1): Enrolled at site two, self-reported Hispanic, had one or more pregnancy complications, had an ARI medical encounter prior to the study, were about 2 years younger (2.5 and 2.1 years at two sites), had a child aged < 13 years in the home, and were earlier in pregnancy (by 21 days, at site two only). However, medical enrollees were less likely than cohort enrollees to: be white, be married, receive the influenza vaccine, have an advanced degree and high subjective social status, and have high self-rated health (Table 1 and Additional file 2: Table S1). In the multivariable model, study site, education, prior ARI medical encounter, pregnancy complications, influenza vaccination, and self-rated health remained independently associated with enrollment type (Additional file 2: Table S1).

### Influenza vaccination differences in year one

The percentage who received the influenza vaccine in the study year among medical enrollees (54%) was similar to the percentage of vaccine uptake among participant refusers (54%) and those never reached (53%), but were considerably lower than among cohort enrollees (76%). After adjusting for all the bivariate differences between the cohort and medical enrollees (Table 1), the odds of being vaccinated with the influenza vaccine was 2.02-fold (95% CI = 1.54–2.65) higher among the cohort enrollees compared with the medical enrollees (Additional file 2: Table S2).

### Medical enrollees vs. controls vs. non-participants in year two

We did not record the number we attempted to contact in year two. Nonetheless, of the women reached, site two enrolled a higher proportion of women (184/469, 39%) than site one (70/299, 23%); the cross-site enrollment rate was 33% (Additional file 1: Figure S2). In year two, non-white women were more likely to enroll (143 of 321 reached, 45%) than white women (120 of 429 reached, 28%). Both medical and control enrollees were more likely to be influenza vaccinated than refusers (Table 2). Compared to all other pregnant women (excluding refusers), year two enrollees were more likely to be non-white, have a chronic medical condition, have at least one pregnancy complication, have medical encounters for ARI during the year prior to the study, and be earlier in their pregnancy (site one only).

### Characteristics associated with participation in follow-up surveillance

Returning to our year one cohort, 80% (1106/1374) of the pregnant women who joined the cohort completed at least one weekly surveillance report; on average, these surveillance participants completed reports during 55% (95% CI = 53–56%) of the surveillance weeks (interquartile range = 38–71%). As shown in Table 3, characteristics associated with both starting surveillance and (among those who started) completing more weekly reports were: enrollment at site one, white race, non-Hispanic ethnicity, being earlier in the pregnancy, and having no children aged < 13 years at home. Starting surveillance was somewhat less common among women aged ≥35 years, but among surveillance participants, the youngest women (aged < 25 years) completed fewer weeks of surveillance. Other characteristics were associated with either starting or completing weekly surveillance, but not both. Specifically, women who started surveillance were more likely to have an ARI medical encounter during the study period than those who failed to start. Completion of a greater percentage of weekly surveillance reports was higher among married women and was lower among women who had three or more stressful events in the prior year. In multivariable analyses, all of these bivariate associations remained statistically significant when entered simultaneously with the exception of the woman’s age which did not maintain an independent association (Additional file 2: Table S3).

**Table 3:** Characteristics of cohort participants who completed at least one surveillance report and among these surveillance participants, the percentage of weekly reports completed

	Cohort Enrollees (Year 1)	Completed One or More Surveillance Reports		Among Surveillance Participants, Percentage (95% CI) of Weeks with Completed Reports ^a^	
	N (Col. %)	N (Row %)	*p*-value ^b^		*p*-value ^c^
Total N	1374 (100)	1106 (80)		55 (53–56)	
Medical Record Variables					
Study Site			*p* < .0005		*p* < .0005
Site 1	565 (41)	503 (89)		63 (61–65)	
Site 2	809 (59)	603 (75)		47 (46–49)	
Race (White)			*p* < .0005		*p* < .0005
White	934 (68)	785 (84)		57 (56–59)	
Non-White	440 (32)	321 (73)		48 (45–50)	
Ethnicity			*p* < .0005		*p* < .0005
Hispanic/Latina	173 (13)	122 (71)		48 (44–52)	
Non-Hispanic	1201 (87)	984 (82)		55 (54–57)	
High Risk Medical Conditions					
Chronic Condition (prior to pregnancy)	277 (20)	228 (82)	*p* = .39	57 (54–60)	*p* = .08
No condition	1097 (80)	878 (80)		54 (52–56)	
Pregnancy Complication	1034 (75)	830 (80)	*p* = .72	54 (53–56)	*p* = .50
No complication	340 (25)	276 (81)		55 (53–58)	
Medical visits for ARI (1 or more)					
Year Prior to Study (12 months)	320 (23)	255 (80)	*p* = .68	55 (53–56)	*p* = .69
No visit	1054 (77)	851 (81)		55 (53–56)	
Study Period (6 months)	307 (22)	234 (76)	*p* = .03	55 (53–56)	*p* = .76
No visit	1067 (78)	872 (82)		55 (53–56)	
Study Season’s Influenza Vaccine (received)	1038 (76)	847 (82)	*p* = .07	55 (52–58)	*p* = .88
Not received	336 (24)	259 (77)		55 (53–56)	
Interview variables, N (Col. %)					
Woman’s Age (at start of season), Years ^d^			*p* = .04		*p* = .04
< 25	112 (8)	93 (83)		49 (44–54)	
25–29	317 (23)	261 (82)		55 (52–58)	
30–34	558 (41)	459 (82)		56 (54–58)	
≥ 35	387 (28)	293 (76)		54 (51–56)	
Gestational Age (at start of season), Days ^d^			*p* < .0005		*p* < .0005
< 137	339 (25)	306 (90)		65 (53–67)	
137–172	363 (26)	283 (78)		56 (54–59)	
173–224	356 (26)	301 (85)		53 (51–55)	
≥ 225	316 (23)	216 (68)		40 (37–43)	
Race (Self-Reported)			*p* < .0005		*p* < .0005
White	934 (68)	785 (84)		57 (56–59)	
Asian	205 (15)	154 (75)		52 (48–55)	
Black	64 (5)	40 (63)		44 (37–51)	
Mixed or Other	171 (12)	127 (74)		44 (40–48)	
Marital Status			*p* = .61		*p* = .003
Married or Partnered	1286 (94)	1037 (81)		55 (54–57)	
Not Married and Not Partnered	88 (6)	69 (78)		47 (41–52)	
Education			*p* = .14		*p* = .08
High school or less	119 (9)	92 (77)		50 (42–54)	
Some college or bachelor’s degree	744 (54)	589 (79)		55 (53–57)	
Advanced degree	511 (37)	425 (83)		55 (53–57)	
Subjective social status			*p* = .23		*p* = .77
1 to 3 (low)	25 (2)	20 (80)		51 (41–61)	
4 to 6 (medium)	697 (51)	555 (80)		55 (53–57)	
7 to 9 (high)	632 (46)	518 (82)		54 (52–56)	
*Missing or refused*	20 (1)	13 (65)		57 (43–71)	
Child(ren) aged < 13 years at home			*p* < .0005		*p* = .001
No	644 (47)	549 (85)		57 (55–59)	
Yes child	730 (53)	557 (76)		52 (51–54)	
Self-rated Health Status			*p* = .11		*p* = .24
Poor, fair, or good	301 (22)	234 (78)		52 (49–55)	
Very good	548 (40)	435 (79)		55 (53–57)	
Excellent	525 (38)	437 (83)		55 (53–54)	
Smoking			*p* = .34		*p* = .68
Never smoked	1016 (74)	824 (81)		55 (53–56)	
Previously or currently smoke	358 (26)	282 (79)		54 (51–57)	
Stressful events in past year (number)			*p* = .11		*p* = .001
0	276 (20)	231 (84)		55 (54–58)	
1	349 (25)	290 (83)		56 (54–59)	
2	310 (23)	243 (78)		57 (55–60)	
3 or more	439 (32)	342 (78)		51 (48–53)	

*Abbreviations*: *CI* Confidence interval, *ARI* Acute respiratory illness

^a^Since some women had delivery dates during the surveillance period, women differ in the number of eligible surveillance weeks; we report the percentage of eligible surveillance weeks when at least one surveillance report was completed

^b^p-value is from Chi-square test of difference between groups in percentage completing at least one surveillance

^c^p-value is from Student’s t-test or F-ratio comparing the mean percentage of weeks when surveillance was completed between groups

^d^Women’s age and age of fetus were calculated using a common reference date, which was the start of the influenza season at each study site; gestational age is reported in quartiles

Nearly half (49%) of the surveillance participants used two or more methods to complete surveillance reports, and this was associated with a higher percentage of weeks with completed reports compared to using a single method (60% [95% CI = 58–61%] vs. 50% [95% CI = 48–52%] of weeks, respectively) (Additional file 2: Table S2). However, women who used the website exclusively and thus did not use any of the telephone-based reporting methods for their surveillance reports had the highest percentage of weeks with completed reports (66%, 95% CI = 64–69%); they were more likely to have the following characteristics (Additional file 2: Table S3): be enrolled at site one, be white, be non-Hispanic, have a chronic medical condition (prior to pregnancy), have an ARI medical encounter during the study, received the influenza vaccine, be earlier in pregnancy, be married, have completed higher education, and have no children aged < 13 years at home. However, in a multivariable model, only study site, influenza vaccination, marital status, and no child aged < 13 years remained independently associated with exclusive use of the website (Additional file 2: Table S3).

### Characteristics of women retained compared to those lost to 6-month post-delivery follow-up

Of the 1726 women enrolled during years one and two (including cohort, medical, and ARI-negative control enrollees), we completed 6-month post-delivery follow-up interviews with 1595 (92%) participants (Table 4). Completion of the follow-up interview was associated with the following characteristics: enrollment at site one, self-reported white or Asian race, non-Hispanic, no ARI medical encounter during the study, influenza vaccination, age ≥ 25 years, being married, higher education, moderate or higher subjective social status, no children aged < 13 years, better self-rated health, and never smoked. In a multivariable model, study site, no ARI medical visit, age, marital status, no child at home, and self-rated health maintained independent associations with completing follow-up (Additional file 2: Table S4).

**Table 4:** Characteristics of all enrollees in year one and year two who completed 6-month post-delivery follow-up interviews

	All Enrollees ^a^ N (Col. %)	Completed 6-Month Follow-up Interview N (Row %)	
Total N	1726 (100)	1595 (92)	
Medical Record Variables			
Study Site			*p* < .0005
Site 1	651 (38)	625 (96)	
Site 2	1075 (62)	970 (90)	
Race (White)			*p* = .047
White	1100 (64)	1027 (93)	
Non-White	626 (36)	568 (91)	
Ethnicity			*p* = .007
Hispanic/Latina	277 (16)	245 (88)	
Non-Hispanic	1449 (84)	1350 (93)	
High Risk Medical Conditions			
Chronic Condition (prior to pregnancy)	347 (20)	326 (94)	*p* = .23
No condition	1379 (80)	1269 (92)	
Pregnancy Complication	1327 (77)	1226 (92)	*p* = .95
No complication	399 (23)	369 (92)	
Medical visits for ARI (1 or more)			
Year Prior to Study (12 months)	358 (21)	324 (91)	*p* = .13
No visit	1368 (79)	1271 (93)	
Study Period (6 months)	393 (23)	352 (90)	*p* = .02
No visit	1333 (77)	1243 (93)	
Study Season’s Influenza Vaccine (received)	1235 (72)	1152 (93)	*p* = .03
Not received	491 (28)	443 (90)	
Interview variables, N (Col. %)			
Woman’s Age (at start of season), Years ^b^			*p* < .0005
< 25	176 (10)	140 (80)	
25–29	412 (24)	382 (93)	
30–34	677 (39)	642 (95)	
≥ 35	461 (27)	431 (93)	
Gestational Age (at start of season), Days ^b^			*p* = .58
< 137	447 (26)	410 (92)	
137–172	437 (25)	406 (93)	
173–224	437 (25)	409 (94)	
≥ 225	405 (23)	370 (91)	
Race (Self-Reported)			*p* = .035
White	1100 (64)	1027 (93)	
Asian	248 (14)	231 (93)	
Black	88 (5)	76 (86)	
Mixed or Other	290 (17)	261 (90)	
Marital Status			*p* < .0005
Married or Partnered	1603 (93)	1496 (93)	
Not Married and Not Partnered	123 (7)	99 (80)	
Education			*p* < .0005
High school or less	178 (10)	151 (85)	
Some college or bachelors degree	955 (55)	879 (92)	
Advanced degree	593 (34)	565 (95)	
Subjective social status			*p* < .0005
1 to 3 (low)	43 (2)	33 (77)	
4 to 6 (medium)	896 (52)	837 (93)	
7 to 9 (high)	763 (44)	705 (92)	
*Missing or refused*	24 (1)	20 (83)	
Child(ren) aged < 13 years at home			*p* = .02
No	786 (46)	739 (94)	
Yes child	940 (54)	856 (91)	
Self-rated Health Status			*p* = .002
Poor, fair, or good	416 (24)	372 (89)	
Very good	693 (40)	658 (95)	
Excellent	617 (36)	565 (92)	
Smoking			*p* = .005
Never smoked	1272 (74)	1189 (93)	
Previously or currently smoke	454 (26)	406 (89)	
Stressful events in past year (number)			*p* = .81
0	325 (19)	300 (92)	
1	438 (25)	409 (93)	
2	369 (21)	338 (92)	
3 or more	594 (34)	548 (92)	

*Abbreviations*: *NA* Not applicable, *ARI* Acute respiratory illness

^a^All enrollees includes cohort enrollees (year 1), medical enrollees (years 1 and 2), and ARI-negative enrollees (year 2)

^b^Women’s age and age of fetus were calculated using a common reference date, which was the start of the influenza season at each study site

### Consistent associations across participation indicators

Study site and race were significantly associated with all cohort participation indicators (cohort recruitment, surveillance participation, and retention); non-Hispanic ethnicity and not having a child aged < 13 years were associated with all indicators where these factors could be examined. Women with additional health issues (as indicated by chronic medical conditions, pregnancy complications, and medical encounters for ARI prior to pregnancy and during the study period) were more likely to be reached for recruitment and more likely to enroll in the cohort. However, among women who were enrolled in the cohort, these health issues were not consistently associated with surveillance participation or study retention. Similarly, pregnant women who received influenza vaccination were more likely to be reached and more likely to enroll in the cohort; yet, among cohort enrollees, vaccination status was not consistently associated with surveillance participation or retention.

## Discussion

We had greater difficulty enrolling cohort participants and conducting active surveillance than we expected [5, 16]. We only reached half of the potential participants we tried to contact by telephone in year one, and only 1 in 5 of the women we invited agreed to join the cohort. Participants clearly differed from non-participants. We found statistically significant differences between enrollees, refusers, and women never reached on all ten of the characteristics we could obtain from medical records. One in 5 cohort enrollees never started their weekly surveillance reports, and among those who did participate, reports were only completed for about half of the weeks. Nonetheless, retention among all enrollees was very high (92%) for a 6-month follow-up interview. Here too, we noted statistically significant associations between surveillance participation, retention, and most of the socio-demographic and health characteristics we examined.

Race was a consistent predictor in all of our analyses. Non-white women were less likely to be reached and enroll in our cohort; once in the cohort, non-white women were less likely to start active surveillance, completed fewer weekly reports, and were less likely to complete the 6-month follow-up interview. This finding is consistent with previous observations of lower rates of completed contacts and research consent by non-white adults [17–19]. However, in year two, when participation did not involve weekly surveillance, non-white women who we reached and invited to participate as a medical or control enrollees were more likely to consent to participate than white women. The difference we observed between years is consistent with reviews that note research participation is not always lower among non-white adults and may depend on study type and tasks [19, 20].

Although the differences we noted were statistically significant, many were of relatively small magnitude. Certainly, statistical differences in participation among subgroups do not necessarily imply that the study findings are biased. In our IVE study, we were most concerned about potential confounders: characteristics associated with both the exposure of interest (vaccination) and the outcome (laboratory-confirmed influenza illness). We previously reported that only gestational age at enrollment was associated with both the likelihood of vaccination and influenza outcomes in our final study sample [6]. However, we noted here that women who were not reached and those who refused cohort participation were less likely to receive the influenza vaccine during pregnancy and were in somewhat better health (as indicated by fewer chronic conditions, pregnancy complications, and medical encounters for ARI). Enrolling a sample that was somewhat less healthy than other pregnant women in the community would not necessarily change the IVE we observed; indeed, there is little evidence that socio-demographic factors (other than age) or underlying health conditions modify IVE [21–23]. Nonetheless, enrolling a cohort with high vaccination coverage likely contributed to the fact that we observed much fewer influenza illnesses than we expected [5], and systematic differences in race and health may have biased our results in unpredictable ways [24].

Enrolling women following medical encounters for ARI proved to be an efficient way to increase the total number of influenza positive cases in our study. The IVE literature typically assumes that ARI patients differ from their full local population in their propensity for medical utilization and vaccination [21, 25]. Indeed, the test-negative design, which compares vaccination rates among influenza positive patients with that of influenza negative patients, is believed to minimize potential biases associated with healthcare seeking behavior by focusing on people who have sought medical care for similar symptoms [21, 25]. However, in study year one, we found that influenza vaccine uptake among medical enrollees was similar to cohort refusers and the large population of pregnant women never reached. In contrast, our cohort participants stood out as having very different influenza vaccine uptake; pregnant women in our cohort were 2-fold more likely to receive the influenza vaccine than medical enrollees even after adjusting for socio-demographic and health characteristics, indicating a higher levels of vaccination compliance among women who participated in the cohort study. This suggests that the medical enrollees in our previously published IVE estimates [6] may have been more representative of the source population of pregnant women in these healthcare systems than we assumed.

Enrolling women following medical encounters also proved to be an effective way in year one of our study to reach and enroll women who were non-white, Hispanic, in poorer health, and had children at home. In year two, non-white women were also more represented among the medical enrollees. This may have been due in part to the fact that we were able to reach a more diverse population of women when many were at home sick. Although we asked the cohort and medical enrollees to perform similar activities (including written informed consent, an enrollment interview, respiratory specimen collection), the commitment asked of medical enrollees was more immediate and clear, since they often completed the bulk of study tasks on the same day. In retrospect, we recognize that the scope of tasks involved in our cohort study, the uncertainty of what would be required (depending on if they became ill or not), and the extended time commitment, may have biased participation toward women who were more educated, had higher subjective social status, had fewer stressful events in the past year, already had health concerns, and were more likely to have future pregnancy and ARI issues.

Among the strengths of our study is the detailed report of participation rates which start with the denominator of all presumed eligible potential participants, as recommended by the Strengthening the Reporting of Observational Studies in Epidemiology (STROBE) guidelines [26], but rarely published as part of cohort study results. Because our study took place within integrated healthcare systems, we were also able to describe summary counts of the demographic and health characteristics of those never reached and cohort refusers; these characteristics are usually unavailable when studies exclude individual-level data on those who refuse research consent. Indeed, greater clarity in reporting on potential selection biases is essential to interpreting previous and future findings regarding the possible benefits of vaccines for pregnant women and their infants [24]. In our study, the association of participant engagement with poor health and influenza vaccination status would have been missed had we focused only on consented enrollees, since the differences we noted were only apparent earlier in the steps of contacting and recruiting participants.

Our study also has at least five limitations. First, we do not have information on the specific reasons why some women refused participation or did not complete surveillance tasks. Second, the extent to which our findings will generalize to other study settings and designs may be limited by the features of our study. For example, our study benefited from being able to offer small financial incentives for participation, from conducting home visits rather than requiring special trips to research facilities, and from having regularly updated contact and medical care information within the integrated healthcare systems. Third, we did not have access to complete information on Hispanic ethnicity or racial categories beyond white vs. non-white for all potential participants, which limited our ability to describe and understand how these factors influenced participation. Fourth, we noted differences in participation between study sites, which may have been due to different recruitment and staffing methods, population differences in race and ethnicity, or other reasons. In year one, study site one used local staff (with local telephone numbers) for recruitment and had higher enrollment and surveillance participation rates than site two which relied on an external contractor. Interestingly, in year two, when both sites used local staff for recruitment, the percentage of contacted women who enrolled was higher at site two. Finally, our surveillance methods did not include the use of short message service (SMS) texts, which have recently been demonstrated to be an effective way to conduct health monitoring during pregnancy (e.g., [27, 28]).

## Conclusions

In conclusion, we observed systematic differences in socio-demographic and health characteristics across different levels of participant engagement and in comparing cohort vs. medical enrollees. The indications of selection bias we observed are a reminder that having a representative sample and a high participation rate remains critically important for ensuring valid conclusions in observational studies. Faced with a growing need to conduct observational studies of pregnant women due to new disease threats [29, 30] and new opportunities for immunization [31–35] and treatments [36] during pregnancy, more methodological research and innovation in conducting prospective observational studies in this population is needed [16], especially when extended participant engagement and ongoing surveillance are required.

## Additional files

Additional file 1: **Figure S1.** Recruitment in Year 1 as Cohort or Medical Enrollees. **Figure S2.** Recruitment in Year 2 as Medical Enrollees or Acute Respiratory Illness (ARI) Negative Controls. (PPTX 40 kb)

Additional file 2: **Table S1.** Multivariable Analyses Including Covariates with Significant Bivariate Associations. Presents full results of logistic regressions models described in the manuscript. **Table S2.** The surveillance methods used and the percentage of weekly reports completed by method or combination of methods. Presented the percentage who completed surveillance reports. **Table S3.** Characteristics of Cohort Participants who Completed Combinations of Computer Automated Telephone Interview (CATI) and Website Methods of Active Surveillance for Acute Respiratory Illness. Presents descriptive characteristics by types of surveillance methods used. (PDF 73 kb)

## Abbreviations

ARI: Acute respiratory illness
CATI: Computer assisted telephone interviewing
EMR: Electronic medical record
ICD-9 CM: International Classification of Diseases, Ninth Revision, Clinical Modification
IVE: Influenza vaccine effectiveness
IVR: Interactive voice response system
SRH: Self-rated health
SSS: Subjective social status
