# Supplementary material for: Factors associated with recruitment, surveillance participation, and retention in an observational study of pregnant women and influenza
Source: BMC Pregnancy Childbirth. 2019 May 8;19:161. doi: 10.1186/s12884-019-2280-0 (PMC6507168; doi:10.1186/s12884-019-2280-0)
Supplement: Supplementary file 1 — Figure S1. Recruitment in Year 1 as Cohort or Medical Enrollees. Figure S2. Recruitment in Year 2 as Medical Enrollees or Acute Respiratory Illness (ARI) Negative Controls. (PPTX 40 kb) [file 12884_2019_2280_MOESM1_ESM.pptx]

## Slide 1
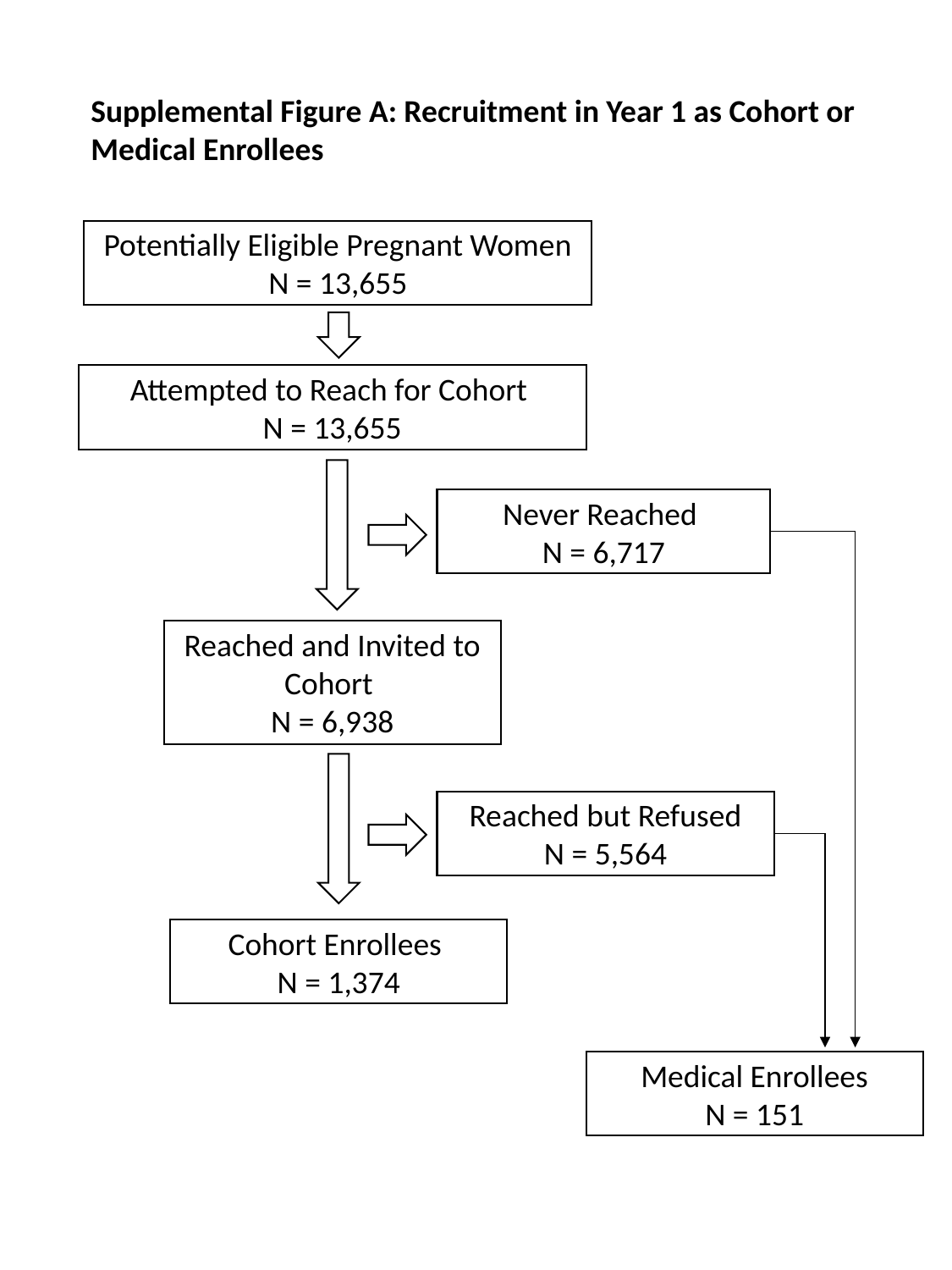

Supplemental Figure A: Recruitment in Year 1 as Cohort or Medical Enrollees
Potentially Eligible Pregnant Women
N = 13,655
Attempted to Reach for Cohort
N = 13,655
Never Reached
N = 6,717
Reached and Invited to Cohort
N = 6,938
Reached but Refused
N = 5,564
Cohort Enrollees
N = 1,374
Medical Enrollees
N = 151

## Slide 2
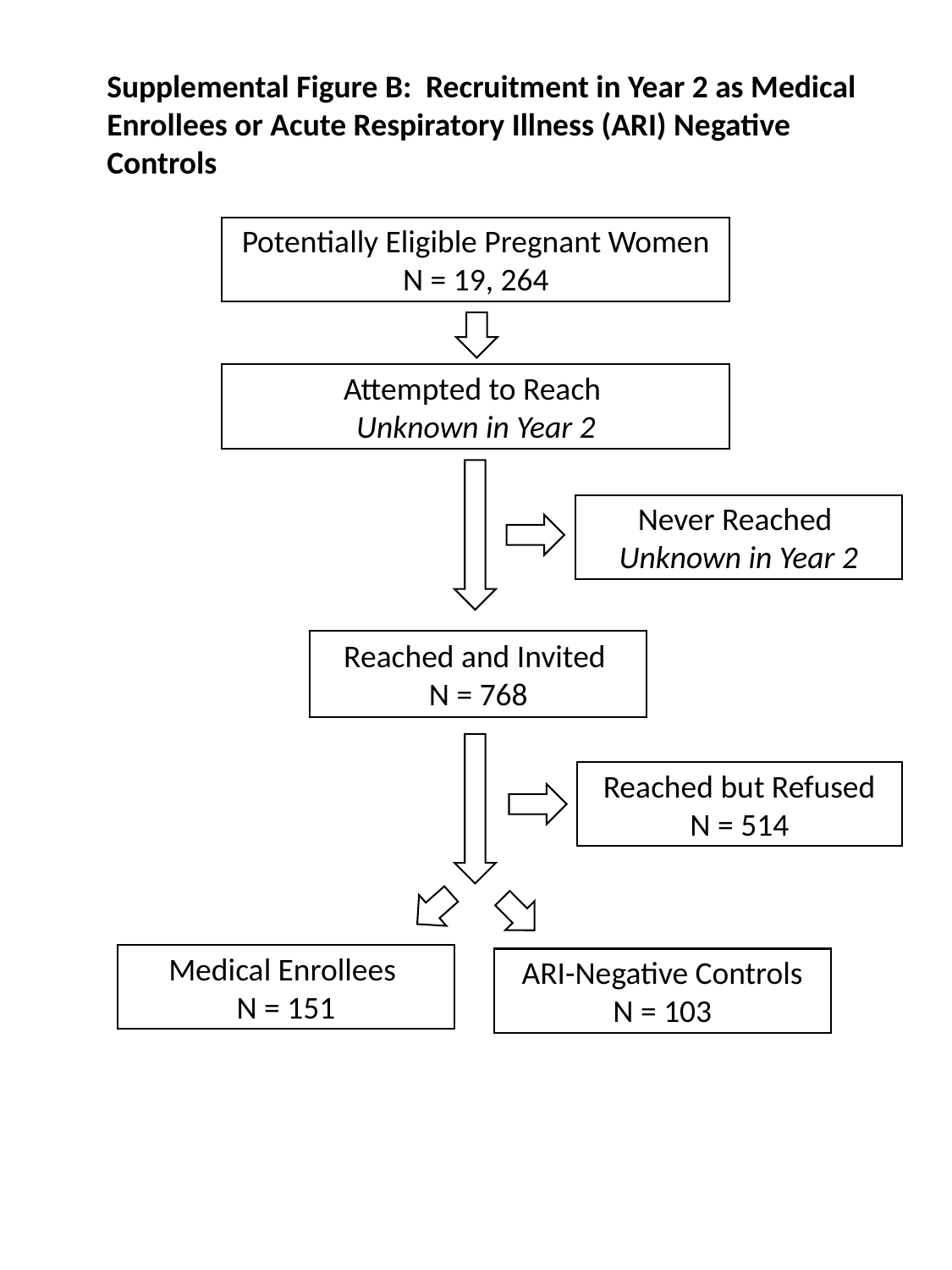

Supplemental Figure B: Recruitment in Year 2 as Medical Enrollees or Acute Respiratory Illness (ARI) Negative Controls
Potentially Eligible Pregnant Women
N = 19, 264
Attempted to Reach
Unknown in Year 2
Never Reached
Unknown in Year 2
Reached and Invited
N = 768
Reached but Refused
N = 514
Medical Enrollees
N = 151
ARI-Negative Controls
N = 103
