# Supplementary material for: Factors associated with recruitment, surveillance participation, and retention in an observational study of pregnant women and influenza
Source: BMC Pregnancy Childbirth. 2019 May 8;19:161. doi: 10.1186/s12884-019-2280-0 (PMC6507168; doi:10.1186/s12884-019-2280-0)
Supplement: Supplementary file 2 — Table S1. Multivariable Analyses Including Covariates with Significant Bivariate Associations. Presents full results of logistic regressions models described in the manuscript. Table S2. The surveillance methods used and the percentage of weekly reports completed by method or combination of methods. Presented the percentage who completed surveillance reports. Table S3. Characteristics of Cohort Participants who Completed Combinations of Computer Automated Telephone Interview (CATI) and Website Methods of Active Surveillance for Acute Respiratory Illness. Presents descriptive characteristics by types of surveillance methods used. (PDF 73 kb) [file 12884_2019_2280_MOESM2_ESM.pdf]

Supplemental Table A. Multivariable Analyses Including Covariates with Significant Bivariate Associations

A. Cohort Enrollees Compared to Medical Enrollees in Year One

Logistic Regression Predicting Medical (vs. Cohort)

	B	S.E.	Wald	df	Sig.	Exp(B)	95% C.I.for EXP(B)	
							Lower	Upper
Study site (two vs. 1)	-1.79	0.33	28.77	1	0.00	0.17	0.09	0.32
White (vs. non-white)	0.37	0.24	2.44	1	0.12	1.45	0.91	2.32
Hispanic (vs. non-Hispanic)	-0.01	0.29	0.00	1	0.99	1.00	0.56	1.76
Pregnancy complication (vs. not)	1.20	0.37	10.33	1	0.00	3.31	1.60	6.88
ARI visit prior to pregnancy (vs. not)	0.76	0.24	10.32	1	0.00	2.15	1.35	3.42
Flu vaccinated (vs. not)	1.05	0.23	20.15	1	0.00	2.86	1.81	4.52
Maternal age (years)	-0.04	0.02	2.81	1	0.09	0.96	0.92	1.01
Gestational age (days)	0.00	0.00	0.84	1	0.36	1.00	0.99	1.00
Married (vs. not)	0.14	0.38	0.13	1	0.72	1.15	0.55	2.41
Education (High School [REF])			7.00	2	0.03			
College	-0.38	0.35	1.17	1	0.28	0.69	0.35	1.36
Advanced Degree	-1.11	0.45	6.04	1	0.01	0.33	0.14	0.80
Subjective social status (Low [REF])			3.15	3	0.37			
Medium	-0.25	0.57	0.19	1	0.66	0.78	0.26	2.38
High	-0.67	0.60	1.25	1	0.26	0.51	0.16	1.66
Missing	-0.13	0.96	0.02	1	0.89	0.88	0.14	5.74
Self-Rated Health (Low [REF])			12.85	2	0.00			
Very Good	-0.69	0.26	6.83	1	0.01	0.50	0.30	0.84
Excellent	-1.01	0.30	11.02	1	0.00	0.37	0.20	0.66
Constant	-0.87	1.01	0.75	1	0.39	0.42		

2. Influenza Vaccination Differences in Year One

Logistic Regression Predicting Influenza Vaccinated (vs. Not)

	B	S.E.	Wald	df	Sig.	Exp(B)	95% C.I.for EXP(B)	
							Lower	Upper
Study site (two vs. 1)	0.01	0.13	0.00	1	0.96	1.01	0.78	1.31
White (vs. non-white)	-0.11	0.13	0.70	1	0.40	0.90	0.70	1.16
Hispanic (vs. non-Hispanic)	0.00	0.16	0.00	1	0.98	1.00	0.73	1.37
Pregnancy complication (vs. not)	-0.10	0.14	0.57	1	0.45	0.90	0.69	1.18
ARI visit prior to pregnancy (vs. not)	0.39	0.15	7.08	1	0.01	1.48	1.11	1.97
Cohort enrollee (vs. Medical)	0.70	0.14	26.35	1	0.00	2.02	1.54	2.64
Maternal age (years)	0.02	0.01	2.13	1	0.14	1.02	0.99	1.04
Gestational age (days)	0.01	0.00	26.21	1	0.00	1.01	1.00	1.01
Married (vs. not)	0.02	0.22	0.01	1	0.94	1.02	0.66	1.57
Education (High School [REF])			16.66	2	0.00			
College	0.28	0.19	2.01	1	0.16	1.32	0.90	1.93
Advanced Degree	0.77	0.23	11.60	1	0.00	2.16	1.39	3.37
Subjective social status (Low [REF])			5.38	3	0.15			
Medium	0.10	0.34	0.09	1	0.77	1.11	0.57	2.14
High	0.13	0.35	0.14	1	0.71	1.14	0.58	2.25
Missing	1.83	0.82	5.00	1	0.03	6.23	1.25	30.94
Child at home (aged <14) (vs. not)	-0.08	0.12	0.48	1	0.49	0.92	0.74	1.16
Self-Rated Health (Low [REF])			1.00	2	0.61			
Very Good	-0.08	0.15	0.33	1	0.57	0.92	0.69	1.23
Excellent	-0.15	0.15	0.99	1	0.32	0.86	0.63	1.16
Stressful events in year (0 [REF])			1.79	3	0.62			
1	0.03	0.17	0.03	1	0.87	1.03	0.73	1.44
2	-0.17	0.18	0.96	1	0.33	0.84	0.59	1.19
3	-0.09	0.17	0.30	1	0.58	0.91	0.66	1.26
Constant	-1.40	0.55	6.46	1	0.01	0.25		

3. Characteristics Associated with Participation in Follow-up Surveillance

Logistic Regression Predicting Starting Surveillance vs. Not

	B	S.E.	Wald	df	Sig.	Exp(B)	95% C.I.for EXP(B)	
							Lower	Upper
Study site (two vs. 1)	0.64	0.17	13.55	1	0.00	1.89	1.35	2.65
White (vs. non-white)	-0.44	0.15	8.26	1	0.00	0.64	0.48	0.87
Hispanic (vs. non-Hispanic)	0.46	0.20	5.22	1	0.02	1.58	1.07	2.33
ARI visit prior to pregnancy (vs. not)	-0.39	0.16	5.71	1	0.02	0.68	0.49	0.93
Maternal age (years)	-0.02	0.02	2.12	1	0.15	0.98	0.95	1.01
Gestational age (days)	-0.01	0.00	23.97	1	0.00	0.99	0.99	1.00
Child at home (aged <14) (vs. not)	-0.48	0.15	10.76	1	0.00	0.62	0.46	0.82
Constant	3.26	0.57	32.83	1	0.00	26.07		

Logistic Regression Predicting Exclusive Use of the Website for Surveillance vs. Other

	B	S.E.	Wald	df	Sig.	Exp(B)	95% C.I.for EXP(B)	
							Lower	Upper
Study site (two vs. 1)	1.88	0.17	129.33	1	0.00	6.55	4.74	9.06
White (vs. non-white)	-0.18	0.18	1.02	1	0.31	0.84	0.59	1.18
Hispanic (vs. non-Hispanic)	0.35	0.26	1.77	1	0.18	1.41	0.85	2.34
Pregnancy complication (vs. not)	-0.11	0.17	0.41	1	0.52	0.90	0.65	1.25
ARI visit prior to pregnancy (vs. not)	0.07	0.17	0.16	1	0.69	1.07	0.77	1.48
Flu vaccinated (vs. not)	0.45	0.17	7.11	1	0.01	1.57	1.13	2.19
Maternal age (years)	0.01	0.02	0.11	1	0.74	1.01	0.98	1.04
Gestational age (days)	0.00	0.00	1.43	1	0.23	1.00	1.00	1.00
Married (vs. not)	-0.95	0.35	7.21	1	0.01	0.39	0.19	0.77
Education (High School [REF])			0.64	2	0.73			
College	0.21	0.28	0.56	1	0.45	1.24	0.71	2.14
Advanced Degree	0.16	0.31	0.26	1	0.61	1.17	0.64	2.12
Child at home (aged <14) (vs. not)	-0.40	0.15	7.45	1	0.01	0.67	0.51	0.89
Constant	-2.53	0.61	17.02	1	0.00	0.08		

4. Characteristics of Women Retained Compared to Those Lost to 6-Month Post-Delivery Follow-up

Logistic Regression Predicting Completed 6-Month Follow-up Interview (vs. Not)

	B	S.E.	Wald	df	Sig.	Exp(B)	95% C.I.for EXP(B)	
							Lower	Upper
Study site (two vs. 1)	1.27	0.25	25.64	1	0.00	3.56	2.18	5.83
White (vs. non-white)	0.13	0.21	0.37	1	0.54	1.14	0.75	1.72
Hispanic (vs. non-Hispanic)	-0.09	0.26	0.13	1	0.72	0.91	0.55	1.51
ARI visit prior to pregnancy (vs. not)	-0.47	0.21	4.97	1	0.03	0.63	0.41	0.95
Flu vaccinated (vs. not)	0.26	0.20	1.60	1	0.21	1.29	0.87	1.93
Maternal age (years)	0.07	0.02	12.22	1	0.00	1.08	1.03	1.12
Married (vs. not)	-0.91	0.30	9.49	1	0.00	0.40	0.23	0.72
Education (High School [REF])			3.31	2	0.19			
College	0.22	0.30	0.52	1	0.47	1.24	0.69	2.23
Advanced Degree	0.62	0.38	2.69	1	0.10	1.85	0.89	3.86
Subjective social status (Low [REF])			6.90	3	0.08			
Medium	0.66	0.44	2.33	1	0.13	1.94	0.83	4.56
High	0.23	0.46	0.26	1	0.61	1.26	0.52	3.08
Missing	-0.26	0.74	0.12	1	0.73	0.77	0.18	3.32
Child at home (aged <14) (vs. not)	0.52	0.20	6.57	1	0.01	0.71	0.59	0.98
Self-Rated Health (Low [REF])			6.59	2	0.04			
Very Good	0.52	0.25	4.32	1	0.04	1.69	1.03	2.76
Excellent	-0.04	0.24	0.02	1	0.88	0.97	0.60	1.55
Smoker (vs. not)	-0.39	0.21	3.51	1	0.06	0.68	0.45	1.02
Constant	-0.98	0.72	1.85	1	0.17	0.38		

Supplemental Table B. The surveillance methods used and the percentage of weekly reports completed by method or combination of methods

	Completed One or More Surveillance Reports N (Col %)	Among Surveillance Participants, Percentage (95% CI) of Weeks with Completed Reports	
Total N	1,106 (100)	55 (53-56)	
Number of Methods Used			p < .0005
Used only one method	566 (51)	50 (48-52)	
Used two or more methods	540 (49)	60 (58-61)	
Surveillance Methods			
Used Internet Website	815 (74)	62 (61-64)	p < .0005
Did Not	292 (26)	33 (31-35)	
Used Telephone CATI	674 (61)	53 (52-54)	p = .004
Did Not	432 (39)	57 (54-60)	
Used Telephone automated IVR	115 (10)	57 (53-61)	p = .27
Did Not	991 (90)	54 (53-56)	
Surveillance Method Combinations			p < .0005
Used neither Web or CATI	112 (10)	30 (27-34)	
Used CATI only	180 (16)	35 (33-37)	
Used Web only	320 (29)	66 (64-69)	
Used Both Web and CATI	494 (45)	60 (58-61)	

Abbreviations: CATI (Computer assisted telephone interviewing delivered by trained staff to ask questions using a structured interview); IVR (interactive voice response is a technology that allows a computer to interact with humans through the use of voice and tones input via their key pad); web (Internet-based website with surveillance questions)

Supplemental Table C. Characteristics of Cohort Participants who Completed Combinations of Computer Automated Telephone Interview (CATI) and Website Methods of Active Surveillance for Acute Respiratory Illness

	Cohort Enrollees (Year 1) N (Col. %)	Combination of Surveillance Methods Used, N (Row %)				p-value
		**Used Neither [a]**	**Used CATI Only**	**Used Website Only**	**Used Both CATI and Website**	
Total N	1,374 (100)	379 (28)	180 (13)	321 (23)	494 (36)	
Medical Record Variables						
Study Site						p < .0005
Site 1	565 (41)	98 (17)	31 (5)	239 (42)	197 (35)	
Site 2	809 (59)	281 (35)	149 (18)	82 (10)	297 (37)	
Race						p < .0005
White	934 (68)	223 (24)	94 (10)	256 (27)	361 (39)	
Non-White	440 (32)	156 (35)	86 (20)	65 (15)	133 (30)	
Ethnicity						p < .0005
Hispanic/Latina	173 (13)	64 (37)	36 (21)	24 (14)	49 (28)	
Non-Hispanic	1,201 (87)	315 (26)	144 (12)	297 (25)	445 (37)	
High Risk Medical Conditions						p = .002
Chronic Condition (prior to pregnancy)	277 (20)	68 (25)	27 (10)	88 (32)	94 (34)	
No condition	1,097 (80)	311 (28)	153 (14)	233 (21)	400 (36)	
						p = .24
Pregnancy Complication	1,034 (75)	287 (28)	141 (14)	249 (24)	357 (35)	
No complication	340 (25)	92 (27)	39 (11)	72 (21)	137 (40)	
Medical visits for ARI (1 or more)						p = .82
Year Prior to Study (12 months)	320 (23)	86 (27)	47 (15)	74 (23)	113 (35)	

No visit	1,054 (77)	293 (28)	133 (13)	247 (23)	381 (36)	
						p = .002
Study Period (6 months)	307 (22)	91 (30)	38 (12)	79 (26)	99 (32)	
No visit	1,067 (78)	288 (27)	142 (13)	242 (23)	395 (37)	
						p = .003
Study Season's Influenza Vaccine (receive	1,038 (76)	276 (27)	120 (12)	251 (24)	391 (38)	
Not received	336 (24)	103 (31)	60 (18)	71 (21)	103 (31)	
Interview variables, N (Col. %)						
Woman's Age (at start of season), Years						p = .16
<25	112 (8)	32 (29)	16 (14)	30 (27)	34 (30)	
25-29	317 (23)	82 (26)	46 (15)	75 (24)	114 (36)	
30-34	558 (41)	137 (25)	67 (12)	135 (24)	219 (39)	
≥35	387 (28)	128 (33)	51 (13)	81 (21)	127 (33)	
Gestational Age (at start of season), Days						p < .0005
<137	339 (25)	44 (13)	21 (6)	126 (37)	148 (44)	
137-172	363 (26)	87 (24)	54 (15)	67 (18)	155 (43)	
173-224	356 (26)	69 (19)	52 (15)	72 (20)	163 (46)	
≥225	316 (23)	179 (57)	53 (17)	56 (18)	28 (9)	
Race (Research Categories)						p < .0005
White	934 (68)	223 (24)	94 (10)	256 (27)	361 (39)	
Asian	205 (15)	64 (31)	31 (15)	33 (16)	77 (38)	
Black	64 (5)	29 (45)	19 (30)	6 (9)	10 (16)	
Mixed or Other	171 (12)	63 (37)	36 (21)	26 (15)	46 (27)	
Marital Status						p = .004
Married or Partnered	1,286 (94)	347 (27)	161 (13)	309 (24)	469 (36)	
Not Married and Not Partnered	88 (6)	32 (36)	19 (22)	12 (14)	25 (28)	
Education						p = .019
High school or less	119 (9)	44 (37)	18 (15)	25 (21)	32 (27)	

Some college or bachelors degree	744 (54)	207 (28)	104 (14)	181 (24)	252 (34)	
Advanced degree	511 (37)	128 (25)	58 (11)	115 (23)	210 (41)	
Subjective social status						p = .015
1 to 3 (low)	25 (2)	6 (24)	7 (28)	5 (20)	7 (28)	
4 to 6 (medium)	697 (51)	209 (30)	80 (11)	176 (25)	232 (33)	
7 to 9 (high)	632 (46)	156 (25)	93 (15)	137 (22)	246 (39)	
Missing or refused	20 (1)	8 (40)	0 (0)	3 (15)	9 (45)	
Child(ren) aged <13 years at home						p < .0005
No	644 (47)	136 (21)	81 (13)	170 (26)	257 (40)	
Yes child	730 (53)	243 (33)	99 (14)	151 (21)	237 (32)	
Self-rated Health Status						p = .028
Poor, fair, or good	301 (22)	91 (30)	45 (15)	69 (23)	96 (32)	
Very good	548 (40)	155 (28)	71 (13)	143 (26)	179 (33)	
Excellent	525 (38)	133 (25)	64 (12)	109 (21)	219 (42)	
Smoking						p = .09
Never smoked	1,016 (74)	275 (27)	122 (12)	238 (23)	381 (38)	
Previously or currently smoke	358 (26)	104 (29)	58 (16)	83 (23)	113 (32)	
Stressful events in past year (number)						p = .24
0	276 (20)	68 (25)	37 (13)	62 (22)	109 (39)	
1	349 (25)	90 (26)	41 (12)	84 (24)	134 (38)	
2	310 (23)	85 (27)	36 (12)	84 (27)	105 (34)	
3 or more	439 (32)	136 (31)	66 (15)	91 (21)	146 (33)	

Abbreviations: NA (not applicable); ARI (Acute respiratory illness); CATI (Computer assisted telephone interviewing); web (Internet-based website)

[a] Includes those who never started surveillance and those who used IVR (interactive voice response) only.
